# Potential of biomarker-based enrichment strategies to identify critically ill patients for emerging cell death interventions

**DOI:** 10.1038/s41418-025-01545-0

**Published:** 2025-07-19

**Authors:** Cyril Willemart, Ruth Seurinck, Tom Stroobants, Samya Van Coillie, Jorien De Loor, Sze Men Choi, Ria Roelandt, Mohan Rajapurkar, Symen Ligthart, Philippe G. Jorens, Dominique D. Benoit, Yvan Saeys, Evelyne Meyer, Eric Hoste, Tom Vanden Berghe

**Affiliations:** 1 Cell Death Signaling Lab, Infla-Med Centre of Excellence, Department of Biomedical Sciences, University of Antwerp, Antwerp, Belgium; 2 VIB-UGent Center for Inflammation Research, Ghent, Belgium; 3 Department of Applied Mathematics, Computer Science and Statistics, Ghent University, Ghent, Belgium; 4 Laboratory of Experimental Medicine and Pediatrics, Infla-Med Centre of Excellence, University of Antwerp, Antwerp, Belgium; 5 Department of Intensive Care Medicine, Antwerp University Hospital, Edegem, Belgium; 6 Department of Biomedical Molecular Biology, Ghent University, Ghent, Belgium; 7 Department of Anaesthesia, Intensive Care and Pain Medicine, General Hospital Maria Middelares, Ghent, Belgium; 8 Department of Nephrology, Muljibhai Patel Society for Research in Nephro-Urology, Nadiad, India; 9 Intensive Care Unit, Department of Internal Medicine and Pediatrics, Ghent University Hospital, Ghent University, Ghent, Belgium; 10 Department of Veterinary and Biosciences, Laboratory of Biochemistry, Ghent University, Merelbeke, Belgium

**Keywords:** Predictive markers, Prognostic markers, Interleukins

## Abstract

Critically ill patients admitted to the intensive care unit (ICU) frequently suffer from sepsis and severe multiple organ dysfunction with underlying widespread cell death. Pyroptosis and ferroptosis are regulated cell death forms that may serve as potential therapeutic targets. Pyroptosis is a major detrimental factor driving sepsis, which typically results in excessive oxidative stress potentially inducing ferroptotic organ injury. Here, we show that ICU patients with simultaneous pyro- and ferroptosis-positive signatures have the lowest survival probability. This is reflected by significantly elevated levels of pyroptosis-related biomarkers interleukin-1 receptor antagonist (IL-1Ra), IL-18, and growth and differentiation factor-15 (GDF15), as well as the ferroptosis-related biomarkers malondialdehyde (MDA) and catalytic iron (Fe_c_). Moreover, combining these biomarkers with IL-1α, IL-6, IL-10, TNF, and chitinase-3-like protein 1 further improves clinical outcome prediction. The daily monitoring of pyro- and ferroptosis signatures reveals potential intervention opportunities, such as anakinra, tadekinig alfa, lead ferroptosis inhibitors, or a combination thereof. In summary, our findings demonstrate that a targeted biomarker panel enables predictive enrichment of ICU patients, paving the way for timely intervention strategies against pyroptosis or ferroptosis.

## Introduction

The intensive care unit (ICU) is a complex environment where patients with a wide array of critical conditions are treated. Among these conditions, sepsis, a dysregulated immune response to infection, and multiple organ dysfunction syndrome (MODS), a progressive dysfunction of multiple organ systems, are recognized as a global health priority [1] and significantly contribute to ICU admissions and mortality [2, 3]. These conditions often coexist and are fundamentally driven by an auto-amplifying loop of cell death and inflammation in the different organs [4]. Despite advances in critical care, a move to more personalized treatment is needed due to the heterogeneity in disease presentation and progression [5, 6]. Therefore, dynamic monitoring of these processes is essential to stratify critically ill patients and to guide novel intervention strategies targeting detrimental cell death forms and/or inflammation [4].

Plasma and serum biomarkers are essential to enhance our understanding and management of critically ill patients. Typically, cytokines, metabolic products, and other molecular indicators offer insights into the pathophysiological processes occurring in the body [7]. In particular, cytokines such as IL-6, IL-10, IL-18, and IL-1Ra play crucial roles in the inflammatory response and have been associated with various outcomes in critically ill patients [8, 9]. An effective immune response relies on a balanced production of pro- (e.g., IL-6) and anti-inflammatory (e.g., IL-10) cytokines to clear pathogens and restore homeostasis while avoiding a cytokine storm that can lead to tissue damage [10, 11]. Overall, removing all septic mediators from the bloodstream of critically ill patients [12] or neutralizing a particular cytokine has shown either no or little beneficial outcomes in the absence of patient stratification [11]. For example, clinical trials targeting specific cytokines, such as anakinra (an IL-1 receptor antagonist), have often yielded disappointing results [13]. However, a growing body of evidence suggests that these trials could be significantly more effective if patients were stratified based on their cytokine profiles before enrollment, so-called predictive enrichment [14, 15]. Identifying subgroups of patients who are more likely to respond to specific interventions is expected to enhance the clinical relevance and success rates of these trials.

Beyond inflammation, various biomarkers have also been explored in the context of regulated cell death (RCD) in critically ill patients [16]. Notably, ferroptosis, pyroptosis, and necroptosis are gaining increasing attention for their roles in organ dysfunction [17]. While RCD is crucial for cellular adaptation to stress, excessive activation of these pathways—triggered by disruptions in the intra- or extracellular environment due to pathogens or physical trauma—can be detrimental, leading to excessive cell death and tissue damage. Each form of RCD, such as pyroptosis and ferroptosis, has distinct mechanisms and molecular signatures. Pyroptosis, for instance, is a caspase-1-mediated form of cell death that leads to the formation of plasma membrane pores and the release of IL-1β and IL-18 [17]. We have shown that simultaneous targeting of IL-1 and IL-18 protects against experimental inflammation and septic shock [18]. Therefore, early detection of a failing endogenous defense against pyroptosis-driven sepsis is critical and can be achieved through monitoring key biomarkers such as IL-1 receptor antagonist (IL-1Ra) [8] and IL-18 binding protein (IL-18BP) [19]. Ferroptosis, another form of RCD, is characterized by iron-dependent lipid peroxidation, with biomarkers such as malondialdehyde (MDA) and catalytic iron (Fe_c_) serving as indicators [8, 20]. We observed that the severity of multiorgan dysfunction and the probability of death within a cohort of ICU patients are associated with levels of free plasma MDA and Fe_c_ [20]. Moreover, blocking ferroptosis protects mice from injury and death in experimental non-septic multiorgan dysfunction, but not in sepsis-induced multiorgan dysfunction [20]. We hypothesize that in the latter case one might need to simultaneously control sepsis along organ injury by considering combination treatments with anakinra, tadekinig alfa, and lead ferroptosis inhibitors. Other biomarkers, such as urinary chitinase-3-like protein 1 (u-CHI3L1) for early detection of (sepsis-induced) acute kidney injury (AKI) [21], and plasma growth differentiation factor-15 (GDF15) for abdominal sepsis [21, 22], are also typically used, along with various others [13].

In this study, we aim to investigate the utility of a comprehensive panel of biomarkers in predicting clinical outcomes in ICU patients. By examining both the early (on ICU admission) and peak levels (during the first seven days) of these biomarkers, we seek to understand their relationships with important clinical endpoints such as mortality, AKI, sepsis, and organ dysfunction as measured by the validated Sequential Organ Failure Assessment (SOFA) score. Additionally, we employ unsupervised clustering techniques to identify distinct patient subgroups based on biomarker profiles, potentially reflecting molecular cell death fingerprints that could inform personalized treatment strategies. Lastly, as a framework for designing future clinical trials aimed at interventions for specific molecular signatures, we also adopt a supervised approach to stratify patients in function of MDA, IL-18, and IL-1Ra dynamics.

Our dual approach of correlation analysis and predictive modeling not only enhances our ability to predict patient outcomes but also deepens our understanding of the underlying disease mechanisms. By integrating biomarker profiling into routine ICU practice, we aim to pave the way for predictive enrichment and effective management strategies, thereby addressing the inherent heterogeneity of critically ill patients.

## Subjects & methods

### Study population

This study is a post-hoc analysis of a previous cohort of 190 patients who have been hospitalized in the ICU of Ghent University Hospital from September 2012 till August 2014. The inclusion and exclusion criteria are summarized in Table S1.

### Ethics, consent, and permissions

This study was approved by the Ethical Committee of the Ghent University Hospital (Belgian registration number of the study: B670201213147) and was conducted following the declaration of Helsinki and adhering to Good Clinical Practice Guidelines. Written informed consent was obtained from all patients or their legally authorized representatives.

### Sample collection, sample handling, and data collection

For an overview of the sample collection, handling, and data collection, we refer to the initial study from De Loor et al. [21]. Briefly, blood samples were collected daily for seven consecutive days. The first blood samples were collected at enrollment (day 1) and subsequent sampling took place daily at 6 am.

### Biomarker measurements

IL-18, IL-1α, IL-1β, IL-1Ra, IL-10, IL-6, GDF15, and TNF levels in plasma (measured in pg/mL) were assessed using validated bead-based multiplex immune assays on a Luminex 200 instrument (Luminex Corporate, Austin, TX, US). Measurements were conducted with blinding to patient outcomes and group membership.

Free plasma MDA was assessed using the N-methyl-2-phenylindole colorimetric assay, as previously described [23]. In brief, 50 μL of the test sample was mixed with a reagent solution containing N-methyl 2-phenylindole, acetonitrile, and methanol. Under optimal temperature and pH conditions, MDA from the sample reacts with N-methyl 2-phenylindole to form a chromogen. The MDA concentration (in μM) in the test sample was determined by comparison with a standard curve.

Plasma Fe_c_ levels (expressed in μmol/L) were determined using a modified version of the bleomycin detectable iron assay, originally described by Gutteridge, Rowley, and Halliwell [24]. In this assay, bleomycin degrades DNA in the presence of catalytic iron, producing a thiobarbituric acid reactive substance. This substance then reacts with thiobarbituric acid to generate a chromogen, the intensity of which was measured at 532 nm using a spectrophotometer. All reagents, except for bleomycin, were pretreated with Chelex 100 (Bio-Rad; #1421253) to prevent iron contamination.

The concentration of CHI3L1 was measured with a sandwich ELISA (DC3L10, R&D Systems, Minneapolis, MN, USA) in both serum and urine using dilutions as described in the initial study from De Loor et al. [21].

### Outcomes

The Sequential Organ Failure Assessment (SOFA) score was calculated daily using the worst levels of platelets count, PaO2/FiO2 ratio, creatinine level, urinary output, bilirubin level, arterial tensions, and Glasgow Coma Scale [25]. In addition, the APACHE II score was calculated daily by the attending physicians using the original APACHE II definition which integrates acute physiological measurements, patient age, and chronic health conditions [26]. The Third International Consensus Definitions for Sepsis and Septic Shock (Sepsis-3) were used for sepsis stage determination [27]. Briefly, patients who received antibiotic, antiviral, or antifungal agents (excluding prophylaxis) two days before or after a specific day combined with a SOFA score ≥ 2 were classified as “sepsis”. The subset of septic patients with a serum lactate level ≥ 2 mmol/L and a vasopressor requirement to maintain a mean arterial pressure (MAP) ≥ 65 mm Hg were classified as “septic shock”. The data for determination of the SOFA score and sepsis stage were extracted from the electronic patient database. The AKI classification was based on the Kidney Disease | Improving Global Outcomes (KDIGO) guidelines [28]. For survival analyses, 30-day survival was assessed without distinguishing between death occurring in the ICU or after transfer to another department.

### Statistical analysis

Biomarkers were log2-transformed, with a pseudo count of 1. Associations between patient characteristics (i.e., age, gender, BMI, and the use of nephrotoxic drugs such as corticosteroids or nonsteroidal anti-inflammatory drugs before or on the first study day) and comorbidities (i.e., diabetes and heart failure) with clinical parameters (i.e., 30-day survival, septic shock on day 1, AKI on day 1, SOFA score (validated score for organ failure) on day 1, APACHE II score (validated score for severity of illness in the ICU) on day 1, need for mechanical ventilation on day 1) were assessed using the Wilcoxon rank-sum test for continuous variables, and the Chi-Square test for categorical variables.

Spearman correlation was used to assess the relationship between biomarkers and SOFA scores. For these analyses, the maximal biomarker concentrations and SOFA scores during the first seven days in the ICU were used. Spearman’s rank correlation coefficients were interpreted as follows: 0–0.1 corresponds to negligible correlation, 0.1–0.39 corresponds to weak correlation, 0.4–0.69 corresponds to moderate correlation, 0.7–0.89 corresponds to strong correlation, and 0.9 or higher indicates very strong correlation [29]. Missing values of SOFA subscores were imputed through rolling mean imputation using the *imputeTS* package [30].

To evaluate the relationships between maximal biomarker levels during the first seven days after ICU admission and 30-day survival, we first compared the levels between survivors and non-survivors using the Wilcoxon rank-sum test. We then conducted Cox proportional hazards analyses to further explore these relationships, adjusting for age, gender, BMI, and comorbidities. These analyses were performed using the *survival* package [31], after verifying the proportional hazards assumption and the linearity of the covariates. Relationships between maximal biomarker levels and AKI incidence or maximal sepsis stage during the first seven days were evaluated using the Wilcoxon rank-sum test and the Kruskal-Wallis test, respectively. If the Kruskal-Wallis test was significant, pairwise comparisons were performed using Dunn’s test with adjustment for multiple comparisons.

The predictive model for survival was based on the maximal biomarker levels recorded during the first three days after ICU admission. The predictive models for SOFA, septic shock, and AKI were based on biomarker measurements taken on the ICU admission day. All predictive models were optimized using a forward variable selection approach to identify the most relevant predictors. The performance of the models was evaluated using the area under the receiver operating characteristic curve (AUC-ROC) for logistic regression and the R^2^ metric for linear regression. These performance metrics were calculated during leave-one-out cross-validation, which was performed using the *caret* and *MLeval* packages [32, 33].

To identify specific cluster profiles of biomarkers, model-based unsupervised clustering of biomarker levels on the day of ICU admission was performed using the *mclust* package [34] on patients with complete biomarker measurements for that day (*n* = 167). Briefly, a principal component analysis was first conducted, followed by a forward selection process using the *clustvarsel* package, guided by the Bayesian Information Criterion (BIC) (Fig. S1) [35]. Clustering was then performed on the three most informative principal components to optimize subgroup differentiation. Subsequently, the biomarker levels between clusters were compared to better understand the cluster profiles, and we performed a Kaplan-Meier analysis to estimate the survival rate per cluster. Cox proportional hazards analyses were used to investigate the association between each cluster and mortality, adjusted for age, gender, BMI, and comorbidities.

In a separate analysis, longitudinal k-means clustering was performed to identify biomarker trajectories using *kml* and *kml3d* [36] on z-normalized biomarker concentrations. The optimal number of clusters was determined using the BIC and Davies Bouldin index. For the longitudinal clustering, we adjusted the data of patients whose maximal IL-1Ra peak occurred later than the day of admission (*n* = 29). Specifically, their data were shifted leftward so that the peak was aligned with day 1. This transformation was necessary to facilitate the longitudinal clustering of IL-1Ra and its correlated biomarkers.

Benjamini-Hochberg p-value adjustment was applied for multiple testing when appropriate. Adjusted p-values were considered significant when inferior to 0.05. All statistical analyses and graphs were performed in R 4.4.3, using the *ggstatsplot* package [37].

## Results

### Population and demographic characteristics

The demographics of the study population are summarized in Table 1. Additionally, infection foci and isolated microorganisms are detailed in Table S2, providing further insight into the nature of infections within the cohort. Patients who survived 30 days were younger than those who did not survive (*p* = 0.05). Patients who were on chronic nonsteroidal anti-inflammatory drug (NSAID) therapy prior to ICU admission had significantly lower total SOFA scores on the first day compared to those not on chronic NSAID therapy (*p* = 0.02). No other patient characteristics were significantly associated with the variables assessed, including 30-day survival, septic shock on the first day, SOFA score on the first day, and AKI on the first day.

**Table 1:** Demographics of the study population.

Characteristics	Total cohort (*n* = 176)
**Age, years**	
Median (IQR)	61 (19)
Min-max range	18-86
**Gender, n (%)**	
Male	107 (61)
Female	69 (39)
**Body mass index (BMI), kg/m2**	
Median (IQR)	24.22 (5.38)
Min-max range	15.62–42.45
**Highest SOFA during ICU**	
Median (IQR)	9 (4)
Min-max range	2–19
**Highest APACHE II during ICU**	
Median (IQR)	27 (7)
Min-max range	6–43
**Sepsis stage on day 1, n (%)**	
No sepsis	58 (33)
Sepsis	61 (35)
Septic shock	31 (18)
No data	26 (15)
**AKI stage on day 1, n (%)**	
No AKI	138 (78)
Stage 1	35 (20)
Stage 2	3 (2)
Stage 3	0 (0)
**Survival at day 30, n (%)**	
Survived	135 (77)
Deceased	41 (23)
**Diabetes, n (%)**	
Yes	12 (7)
No	164 (93)
**Corticosteroids (chronic), n (%)**	
Yes	22 (12)
No	154 (88)
**NSAID (chronic), n (%)**	
Yes	10 (6)
No	166 (94)
**Mechanical ventilation on day 1, n (%)**	
Yes	117 (66)
No	59 (34)
**Time in the hospital, days**	
Median (IQR)	21 (30)
Min-max range	1–492
**Reason for hospital admission**	
Elective surgery	21 (12)
Medical	107 (61)
Urgent surgery	48 (28)
**Referred from**	
Other hospital	37 (21)
Emergency room	74 (42)
Operating room	18 (10)
Floor	47 (27)

*IQR* interquartile range, *AKI* acute kidney injury, *SOFA* sequential organ failure assessment, *NSAID* non-steroidal anti-inflammatory drug, *APACHE II* Acute Physiology and Chronic Health Evaluation II.

### Targeted biomarker panel improves clinical outcome prediction

**Fig. 1:**
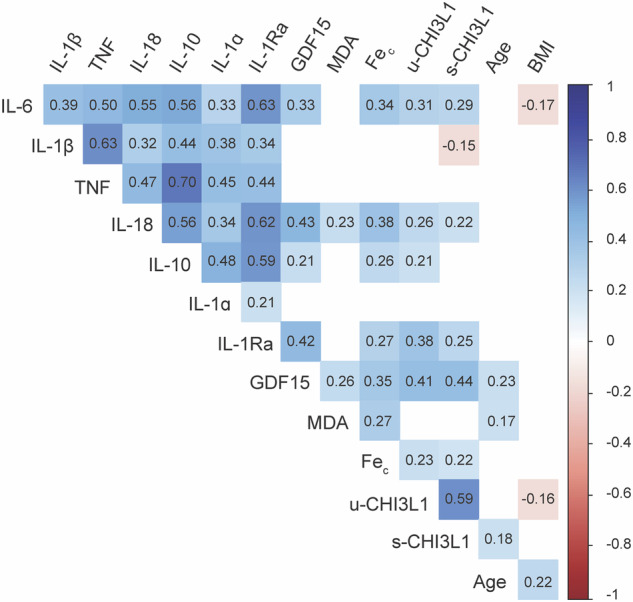
Correlation matrix illustrating the relationships between the maximal value of biomarkers during the first seven days in the ICU, patient ages, and patient BMIs. Spearman’s correlation coefficients are depicted with nonsignificant (*p* > 0.05) correlations removed.

We examined the relationships between the maximum values of various biomarkers during the first seven days of ICU admission, as well as the age and BMI of the patients (Fig. 1). Numerous correlations were identified among these variables, with only the most relevant ones summarized here. Regarding the ferroptosis biomarkers, MDA only correlated weakly with Fe_c_ and IL-18, while Fe_c_ was weakly correlated with IL-18. Regarding the pyroptosis markers, IL-1Ra, IL-18, and GDF15 moderately correlated with each other. Additionally, IL-1Ra and IL-18 moderately correlated with TNF and weakly correlated with both u-CHI3L1 and serum CHI3L1 (s-CHI3L1). BMI showed weak correlations with IL-6, u-CHI3L1, and patient’s age. Age weakly correlated with GDF15, MDA, and s-CHI3L1.

**Fig. 2:**
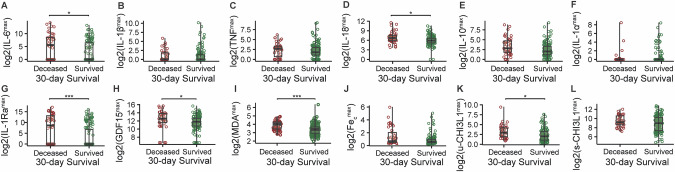
Comparison of maximal biomarker levels during the first seven days in the ICU between patients who survived at least 30 days, and those who did not. **A–L** Boxplots of log2-transformed values of interleukin-6 (IL6), interleukin-1 beta (IL-1β), tumor necrosis factor (TNF), interleukin-18 (IL-18), interleukin-10 (IL-10), interleukin-1 alpha (IL-1α), interleukin-1 receptor antagonist (IL-1Ra), GDF15, free malondialdehyde (MDA), catalytic iron (Fe_c_), urinary chitinase-3-like protein 1 (u-CHI3L1), and serum chitinase-3-like protein 1 (s-CHI3L1) (highest value during first seven days after ICU admission) in patients who deceased within 30 days, and those who survived 30 days. Comparisons between the two groups (deceased: *n* = 41, survived: n = 135) were performed using the Wilcoxon rank-sum test. *: *p* ≤ 0.05, **: *p* ≤ 0.01, ***: *p* ≤ 0.001.

When comparing the maximal biomarker concentration per patient during the first seven days in the ICU, median levels of IL-6, u-CHI3L1, GDF15, IL-18, IL-1Ra, and MDA were significantly higher in the patients who died within 30 days, compared to the patients who survived at least 30 days (Fig. 2). An additional Cox proportional hazards analysis, adjusted for the patient’s age, gender, BMI, and comorbidities, further supports the prognostic value of IL-1Ra and MDA (Table S3). However, after further adjusting for the highest SOFA (Table S4) or APACHE II (Table S5) score reached within the first seven days of ICU admission, none of the biomarkers remained significant, suggesting a strong association between IL-1Ra, MDA, and severity scores based on physiological parameters. Indeed, several biomarkers showed positive correlations with the maximal SOFA score (Fig. S2). TNF, IL-6, IL-18, IL-10, MDA, and u-CHI3L1 exhibited a weak correlation, whereas GDF15, IL-1Ra, Fe_c_, and s-CHI3L1 showed a moderate correlation. The maximal APACHE II score displayed weaker correlations with these biomarkers (Fig. S3).

**Fig. 3:**
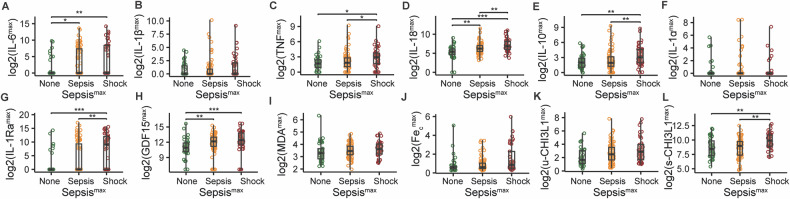
Comparison of maximal biomarker levels during the first seven days in the ICU between patients who experienced different sepsis stages during their ICU stay. **A–L** Boxplots of log2-transformed values of interleukin-6 (IL6), interleukin-1 beta (IL-1β), tumor necrosis factor (TNF), interleukin-18 (IL-18), interleukin-10 (IL-10), interleukin-1 alpha (IL-1α), interleukin-1 receptor antagonist (IL-1Ra), GDF15, free malondialdehyde (MDA), catalytic iron (Fe_c_), urinary chitinase-3-like protein 1 (u-CHI3L1), and serum chitinase-3-like protein 1 (s-CHI3L1). Differences between the three groups (no sepsis: *n* = 38, sepsis: *n* = 77, septic shock: *n* = 41) were assessed using the Kruskal-Wallis test. If significant, pairwise comparisons were performed using Dunn’s test with adjustment for multiple comparisons. *: *p* ≤ 0.05, **: *p* ≤ 0.01, ***: *p* ≤ 0.001.

Additionally, we compared the peak biomarker concentrations between patients who did not experience sepsis, those who experienced sepsis without shock, and those who experienced septic shock within the seven days following their ICU admission (Fig. 3). Median values of IL-6, IL-18, and GDF15 were significantly higher in the sepsis and septic shock groups, compared to the non-sepsis group. TNF, IL-18, IL-10, IL-1Ra and s-CHI3L1 were significantly higher in the septic shock group, compared with the sepsis and non-sepsis groups.

**Fig. 4:**
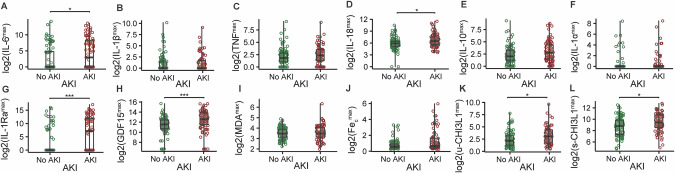
Comparison of maximal biomarker levels between patients who experienced acute kidney injury (AKI) during the first seven days in the ICU and those who did not. **A–L** Boxplots of log2-transformed values of interleukin-6 (IL6), interleukin-1 beta (IL-1β), tumor necrosis factor (TNF), interleukin-18 (IL-18), interleukin-10 (IL-10), interleukin-1 alpha (IL-1α), interleukin-1 receptor antagonist (IL-1Ra), GDF15, free malondialdehyde (MDA), catalytic iron (Fe_c_), urinary chitinase-3-like 1 protein (u-CHI3L1), and serum chitinase-3-like protein 1 (s-CHI3L1) (highest value during first seven days after ICU admission) in patients who experienced AKI during the first seven days after ICU admission and those who did not. Comparisons between the two groups (AKI: *n* = 77, No AKI: *n* = 99) were performed using the Wilcoxon rank-sum test. *: *p* ≤ 0.05, **: *p* ≤ 0.01, ***: *p* ≤ 0.001.

Lastly, we compared the maximal concentration of the biomarkers between patients who were diagnosed with AKI during the first seven days in the ICU and those who were not (Fig. 4). Median values of IL-6, IL-18, IL-1Ra, GDF15, u-CHI3L1, and s-CHI3L1 were significantly higher in the group with AKI, compared with the group without AKI.

**Fig. 5:**
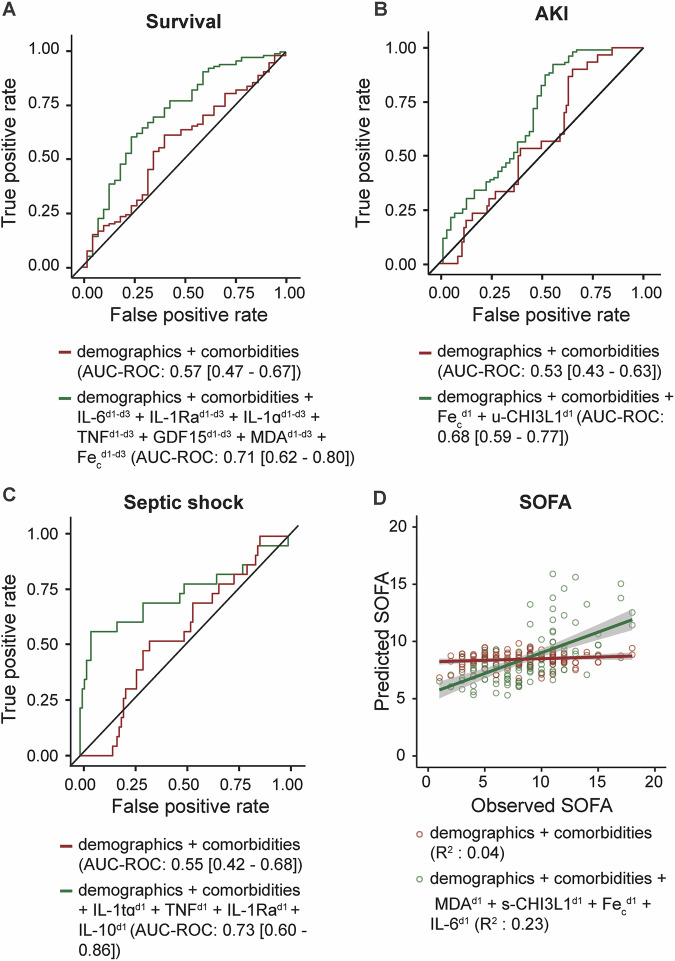
Comparison of outcome predictions when complementing demographics and comorbidities with biomarker levels. **A** ROC curves for survival prediction comparing two models: one including demographics data and comorbidities (red), and an optimized model with additional variables, specifically maximal values during the first three days after ICU admission of a subset of biomarkers (green). **B** ROC curves for acute kidney injury (AKI) prediction comparing a model including demographics data and comorbidities (red), and an optimized model with additional variables, specifically the level on the first day in the ICU of a subset of biomarkers (green). **C** ROC curves for septic shock prediction comparing a model including demographics data and comorbidities (red), and an optimized model with additional variables, specifically the level on the first day in the ICU of a subset of biomarkers (green). **D** Observed SOFA score vs. predicted SOFA score. A linear model using demographics and comorbidities (red) is compared with an optimized model with additional variables, specifically the level on the first day in the ICU of a subset of biomarkers (green).

Next, we aimed to assess the predictive value of the biomarkers for different clinical outcomes, including survival, AKI, septic shock, and SOFA scores. To do this, we compared a restricted model that included patient characteristics and comorbidities with a model where a selection of biomarker levels on the first day (for AKI, SOFA, and septic shock prediction) or maximal levels of the first three days (for survival prediction) were added. Survival prediction without biomarkers yielded an AUC-ROC of 0.57 (95% CI: 0.47–0.67), whereas the AUC-ROC of the optimized model significantly improved to 0.71 (95% CI: 0.62–0.80) (Fig. 5A). When adjusting for the established sepsis diagnosis, the difference between the model with and without biomarkers is no longer significant (Fig. S4). AKI prediction without biomarkers yielded an AUC-ROC of 0.53 (95% CI: 0.43–0.63), whereas the AUC-ROC of the optimized model significantly improved to 0.68 (95% CI: 0.59–0.77) (Fig. 5B). Septic shock prediction without biomarkers yielded an AUC-ROC of 0.55 (95% CI: 0.42–0.68), whereas the AUC-ROC of the optimized model significantly improved to 0.73 (95% CI: 0.60–0.86) (Fig. 5C). Lastly, SOFA score prediction without biomarkers displayed an R^2^ of 0.04 (CV RMSE: 47%), whereas the optimized model yielded an R^2^ of 0.23 (CV RMSE: 39%) (Fig. 5D).

### Unsupervised clustering of biomarkers upon ICU admission identifies a subgroup of patients with poorer survival probability

**Fig. 6:**
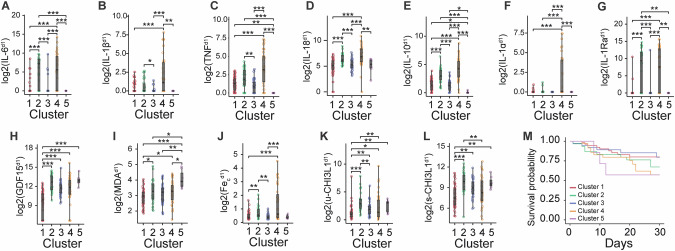
Comparison of day 1 biomarker levels between biomarker-based clusters of patients. **A–L** Boxplots of log2-transformed values of interleukin-6 (IL6), interleukin-1 beta (IL-1β), tumor necrosis factor (TNF), interleukin-18 (IL-18), interleukin-10 (IL-10), interleukin-1 alpha (IL-1α), interleukin-1 receptor antagonist (IL-1Ra), GDF15, free malondialdehyde (MDA), catalytic iron (Fe_c_), urinary chitinase-3-like protein 1 (u-CHI3L1), and serum chitinase-3-like protein 1 (s-CHI3L1) (value on the first day in the ICU) between biomarker-based clusters of patients. **M** Kaplan-Meier survival curves of the clusters during the 30 first days in the hospital. Differences between the three groups comparisons (cluster 1: *n* = 56, cluster 2: *n* = 30, cluster 3: *n* = 39, cluster 4: *n* = 35, cluster 5: *n* = 7) were assessed using the Kruskal-Wallis test. If significant, pairwise comparisons were performed using Dunn’s test with adjustment for multiple comparisons. *: *p* ≤ 0.05, **: *p* ≤ 0.01, ***: *p* ≤ 0.001.

To examine the high-dimensional dataset, we employed an unsupervised clustering technique utilizing Gaussian mixtures with biomarker levels on the admission day as clustering variables. This approach identified five clusters, the primary characteristics of which are outlined in Table 2. Notable differences in biomarker levels between these clusters can be observed (Fig. 6).

**Table 2:** Schematic summary for each biomarker-based cluster.

	Cluster 1	Cluster 2	Cluster 3	Cluster 4	Cluster 5
Hallmark	Other	Ferroptosis ++ Pyroptosis + Sepsis ++	Sepsis +	Ferroptosis ++ Pyroptosis ++ Sepsis ++ AKI +	Ferroptosis + Sepsis +
Biomarker profile	Low IL-6, IL-1ß, IL-18, IL-1ɑ, IL-1Ra, GDF15, MDA, Fe_c_, urinary and serum CHI3L1.	High IL-6, TNF, IL-18, IL-10, IL-1Ra, GDF15, MDA, Fe_c_, urinary and serum CHI3L1; low IL-1ɑ and IL-1ß.	High IL-6, GDF15, urinary and serum CHI3L1; low IL-6, IL-1ß, TNF, IL-18, IL-10, IL-1ɑ, IL-1Ra, MDA, and Fe_c_.	High IL-6, IL-1ß, TNF, IL-18, IL-10, IL-1ɑ, IL-1Ra, GDF15, Fe_c_.	High GDF15, MDA, and serum CHI3L1.
Subject per cluster	n = 56	n = 30	n = 39	n = 35	n = 7
30-day mortality	16%	30%	15%	34%	43%
Max SOFA^d1-d7^ (median (IQR))	8 (4)	11 (3)	8 (5)	11 (4)	11 (3)
Sepsis without shock^d1-d7^	43%	33%	54%	37%	71%
Septic shock^d1-d7^	16%	33%	15%	34%	14%
AKI^d1-d7^	28%	53%	28%	66%	43%

*Fe*_c_ catalytic iron, *SOFA* sequential organ failure assessment, *IQR* interquartile range.

Clusters 2 and 4 exhibited the highest concentrations of IL-18, IL-10, IL-1Ra, and Fe_c_. Cluster 2 was further characterized by high levels of MDA and both urinary and serum CHI3L1 while showing low concentration of IL-1α. In contrast, cluster 4 displayed high concentrations for all interleukins, along with TNF and, overall, the highest variability across all assessed biomarkers. Cluster 1 had the lowest biomarker concentrations across all assessed biomarkers, except for TNF and IL-10.

Although Kaplan-Meier survival curves suggested a lower survival rate in cluster 4 and 5 (Fig. 6M), this trend was not statistically supported by Cox proportional hazards analysis adjusted for patient demographics and comorbidities, with (Table S6) or without (Table S7) adjusting for the SOFA score on the first day.

### Patient stratification for future pyroptosis- & ferroptosis-targeted interventions

To advise the design of future clinical trials targeting specific molecular signatures, we also employed a supervised predictive enrichment approach based on the level of MDA, IL-18, and IL-1Ra, which requires a biomarker-specific approach. We aimed to determine whether an absolute threshold or an estimation of its dynamics (through longitudinal k-means clustering) would be more effective for classifying patients. For MDA, stratifying patients based on an absolute threshold of 14.25 µM—the local minimum of the bimodal distribution for deceased patients, as determined in our previous work [20] —revealed that those with a peak above this threshold had a significantly lower survival probability. However, this significant difference was no longer observed when stratification was based on dynamic changes in MDA concentration (Fig. S5). Conversely, for IL-18, using an absolute threshold did not yield significant differences in survival (Fig. S6A). In contrast, analyzing the dynamics of IL-18 levels—whether increasing or decreasing—showed a significant difference between the two groups (Fig. S6B). Since IL-1Ra levels typically present as a transient peak, an absolute threshold was sufficient, although longitudinal clustering of the joint IL-1Ra/IL-18 trajectories was still feasible (Fig. S7).

**Fig. 7:**
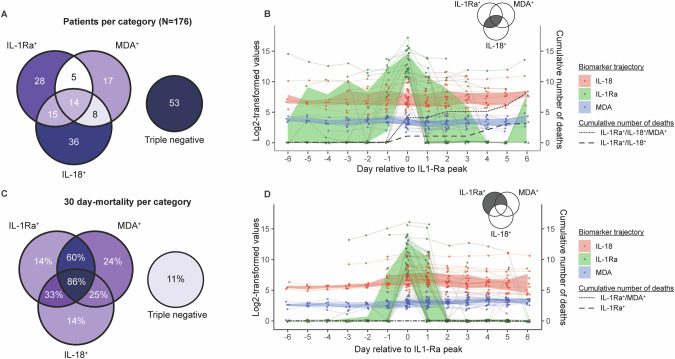
Supervised clustering for targeted intervention. **A** Number of patients per category. **B** 30-days mortality per category. **C** Dynamic of IL-18, IL-1Ra, and MDA in IL-1Ra + /IL-18- patients. **D** Dynamic of IL-18, IL-1Ra, and MDA in IL-1Ra + /IL-18+ patients. Patients are considered IL-1Ra-positive if IL-1Ra was detected during the first 7 ICU days; IL-18-positive if IL-18 peaked above 100 pg/mL detected during the first 7 ICU days; and MDA-positive if MDA peaked above 14.25 µM during the first 7 ICU days.

Consequently, we opted to proceed using the longitudinal clustering for the definition of IL-18-positivity and using the absolute thresholds for MDA- and IL-1Ra-positivity. Using this classification, the triple-positive patients had the highest 30-day mortality rate at 86% (12 out of 14 patients). This was followed by patients who were positive for both IL-1Ra and MDA but negative for IL-18, with a 60% mortality rate (3 out of 5 patients), and those positive for both IL-1Ra and IL-18 but negative for MDA, with a 33% mortality rate (5 out of 15 patients). The largest group, apart from the triple-negative patients who had an 11% mortality rate (6 out of 53 patients), consisted of single-positive IL-18 patients, who had a 14% mortality rate (5 out of 36 patients) (Fig. 7). To further characterize the clinical progression of the triple-positive subgroup, we visualized individual trajectories of IL-1Ra, IL-18, and MDA, alongside daily SOFA scores over the first seven ICU days (Fig. S8). In summary, this analysis suggests that pyroptosis- and ferroptosis-associated biomarker profiles may be linked to survival outcomes, highlighting the need for further mechanistic studies to confirm their causal role. This predictive enrichment approach could pave the way for timely intervention strategies for ferroptosis or pyroptosis.

## Discussion

In this study, we demonstrated that incorporating a diverse array of biomarkers, including cytokines, MDA, CHI3L1, and Fe_c_ levels, significantly enhances the prediction of clinical outcomes in patients admitted to the ICU. Our analysis not only examined the relationships between these biomarkers and various clinical endpoints, such as survival, sepsis, and AKI, but also employed unsupervised clustering to identify distinct patient profiles based on biomarker levels measured on the day of ICU admission.

The correlation analysis revealed that higher peak levels of biomarkers such as IL-6, TNF, IL-18, IL-10, IL-1Ra, GDF15, MDA, u-CHI3L1, and s-CHI3L1 during the first seven days of ICU admission were associated with poorer outcomes, including increased mortality, higher SOFA scores, and a higher incidence of sepsis/septic shock and AKI. These findings highlight the strong link between elevated biomarker levels and the progression of severe clinical conditions, suggesting that early biomarker profiling can be instrumental in identifying high-risk patient trajectories. Importantly, our predictive models showed that incorporating biomarker data significantly improved outcome prediction compared to models based solely on patient demographics and comorbidities. For instance, adding these biomarkers improved the accuracy of survival prediction from an AUC-ROC of 0.57 to 0.71 and septic shock prediction from an AUC-ROC of 0.55 to 0.74. When adjusting the survival prediction model for established sepsis diagnosis, the predictive advantage of biomarkers was no longer statistically significant, highlighting the strong prognostic value of sepsis staging in critically ill patients. These findings suggest that biomarker profiling may be particularly useful for stratifying patients at risk of septic shock and guiding therapeutic interventions, rather than improving mortality prediction beyond sepsis classification. Overall, these findings suggest that early biomarker profiling could help identify patient trajectories and inform timely intervention strategies, although causality cannot be inferred from these associations.

The unsupervised clustering analysis identified five distinct patient clusters based on biomarker levels on the admission day. Cluster 2 and 4, characterized by high levels of Fe_c_ and MDA, exhibited some features of a ferroptosis fingerprint. Cluster 2 was further distinguished by elevated IL-1Ra levels but low levels of IL-1α and IL-1β. Rather than reflecting an overt cytokine storm, this pattern may indicate a preceding IL-1 peak, an incipient high IL-1β concentration, or an immune system imbalance, potentially contributing to the increased mortality and incidence of septic shock observed in this cluster.

Cluster 4 was marked by a highly variable biomarker profile but also exhibited the highest concentrations of both IL-18 and IL-1β, consistent with a pyroptosis-associated signature suggestive of severe inflammasome-driven inflammation. Although these clusters can be linked to specific cell death pathways, they do not directly correlate with survival differences. Instead, only patient age remained significantly associated with survival outcomes. This is not entirely unexpected, as the presence of distinct cell death mechanisms alone does not necessarily determine prognosis. Rather than replacing SOFA, our approach aims to complement existing scoring systems by enabling patient stratification for targeted interventions, ensuring that treatments are directed to those most likely to benefit. These targeted biomarkers enable predictive enrichment of patients eligible for specific treatments.

Our previous research has highlighted the therapeutic potential of simultaneously neutralizing IL-1 and IL-18 in experimental mouse models of septic shock [18], underscoring the relevance of this approach for clinical applications. Building on this, the current study provides new evidence that monitoring both IL-18 and IL-1Ra could be an effective strategy for predictive enrichment in clinical settings. Specifically, we found that patients who were positive for both IL-18 and IL-1Ra had a significantly higher 30-day mortality rate compared to those positive for only one of these markers. Further stratification based on MDA positivity revealed that triple-positive patients (IL-18, IL-1Ra, and MDA) had the highest 30-day mortality rate at 86%, indicating a subgroup with a particularly poor prognosis. This suggests that targeting multiple pathways, such as combining IL-18 and IL-1 blocking strategies with ferroptosis inhibitors [20], could offer a synergistic therapeutic benefit for these high-risk patients. Specifically, treatment with anakinra and/or tadekinig alfa could restore the drop or defect in endogenous production of IL-1Ra and/or IL-18BP, while ferroptosis inhibitors would instantly block lipid peroxidation-driven organ injury.

Our study has some limitations. First, the post-hoc nature of the analysis and the single-center study design may limit the generalizability of our results. Second, differences in medical decision-making, particularly regarding the intensity of life-sustaining treatments, may act as confounders. For example, younger patients may receive more aggressive life-sustaining interventions, whereas treatment limitations may be applied earlier in older patients, even when their overall prognosis is similar. While age was included as an adjustment variable in Cox regression, such differences in care preferences or practices are difficult to fully capture in retrospective analyses. Additionally, while we identified significant correlations between biomarker levels and clinical outcomes, causality cannot be inferred, a common issue in sepsis research [38]. A notable limitation is the lack of detection of IL-18 bound to soluble IL-18BP, which could affect the accuracy of our IL-18 measurements. Similarly, the absence of IL-1R2 measurements might lead to an underestimation of the anti-inflammatory response in certain patients. Future multicenter studies with larger cohorts and more comprehensive biomarker profiling are needed to validate our findings and explore the mechanistic pathways underlying the observed associations.

In conclusion, our study highlights the potential value of incorporating a selected panel of biomarkers into clinical prediction models for ICU outcomes of critically ill patients. By identifying distinct patient clusters based on early biomarker profiling, we provide a foundation for more personalized treatment approaches, particularly in clinical trials focused on targeting the IL-18/IL-1 pathway as well as on ferroptosis inhibition. This research supports the integration of comprehensive biomarker profiling into routine ICU management to improve patient outcomes. Future research should focus on validating these findings across multiple centers and developing practical stratification tools for clinical use.

## Supplementary information

Supplementary

## Data Availability

All the R coding used for data analysis can be found at: https://github.com/tramelliwe/baki2024.
