# Supplementary material for: Potential of biomarker-based enrichment strategies to identify critically ill patients for emerging cell death interventions
Source: Cell Death Differ. 2025 Jul 19;32(12):2284–93. doi: 10.1038/s41418-025-01545-0 (PMC12669244; doi:10.1038/s41418-025-01545-0)
Supplement: Supplementary file 1 — Supplementary [file 41418_2025_1545_MOESM1_ESM.docx]

**Potential of biomarker-based enrichment strategies to identify critically ill patients for emerging cell death interventions**

Cyril Willemart, Ruth Seurinck, Tom Stroobants, Samya Van Coillie, Jorien De Loor, Sze Men Choi, Ria Roelandt, Mohan Rajapurkar, Symen Ligthart, Philippe G Jorens, Dominique D. Benoit, Yvan Saeys, Evelyne Meyer, Eric Hoste, Tom Vanden Berghe

Table of Contents

[Supplementary Figures 2](#_Toc193186596)

[Supplementary Tables 12](#_Toc193186597)

# Supplementary Figures


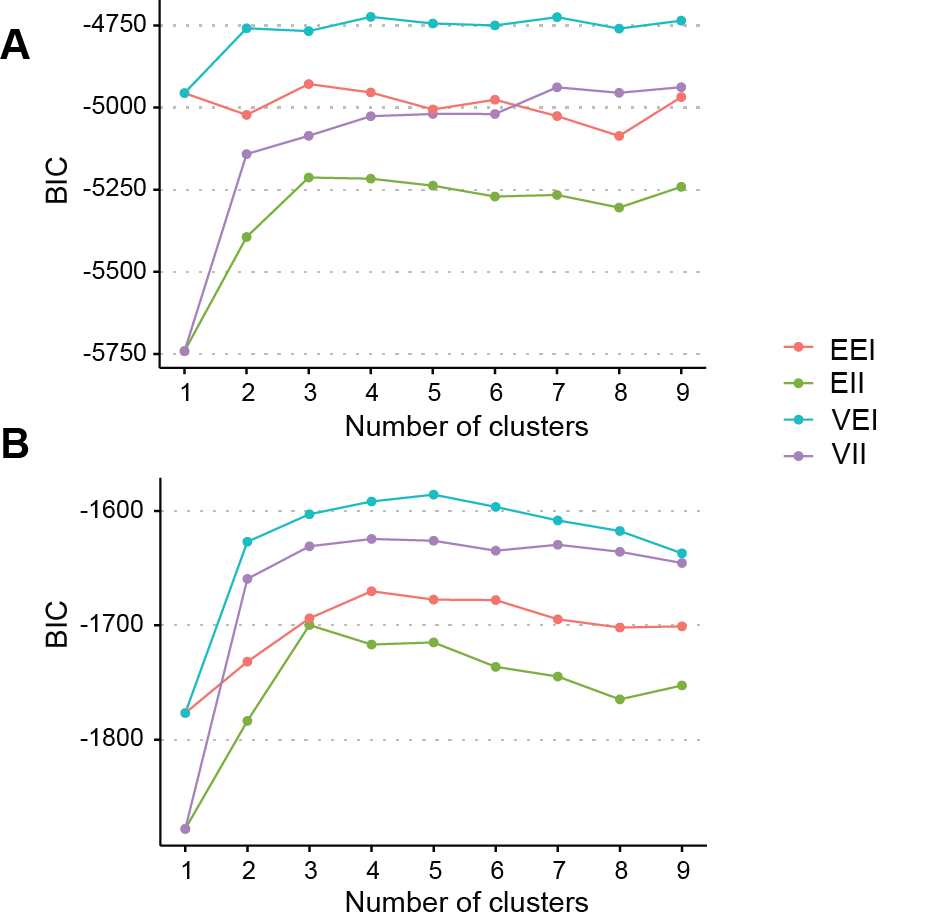


**Figure S1: Bayesian Information Criterion (BIC) as a function of the number of clusters for the model-based unsupervised clustering of biomarker levels on the admission day.**

(A) BIC for the model including all principal components; (B) BIC for the model including the three most informative principal components. The model nomenclature follows the conventions of the *mclust* software, where E and V denote equal and variable characteristics across groups, respectively, and I represents the identity matrix.


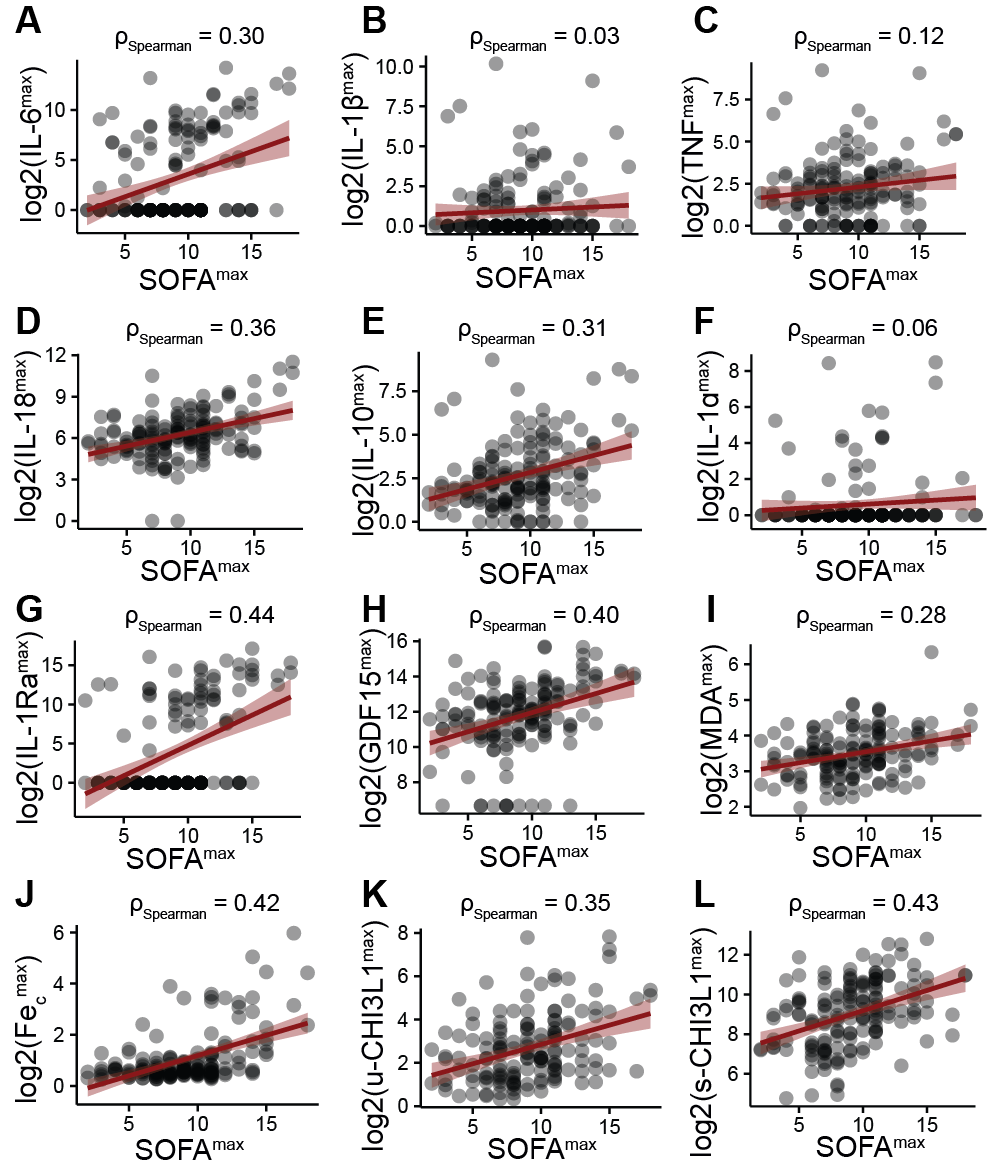


**Figure S2: Scatter plots and correlations between maximal biomarkers levels and SOFA scores during the first seven days in the ICU.**

(A-L) Correlation between log2-transformed values of interleukin-6 (IL6), interleukin-1 beta (IL-1β), tumor necrosis factor (TNF), interleukin-18 (IL-18), interleukin-10 (IL-10), interleukin-1 alpha (IL-1α), interleukin-1 receptor antagonist (IL-1Ra), GDF15, free malondialdehyde (MDA), catalytic iron (Fec), urinary chitinase-3-like protein 1 (u-CHI3L1), and serum chitinase-3-like protein 1 (s-CHI3L1) (highest value during the first seven days after ICU admission) and maximal SOFA score attained during the first seven days after ICU admission.


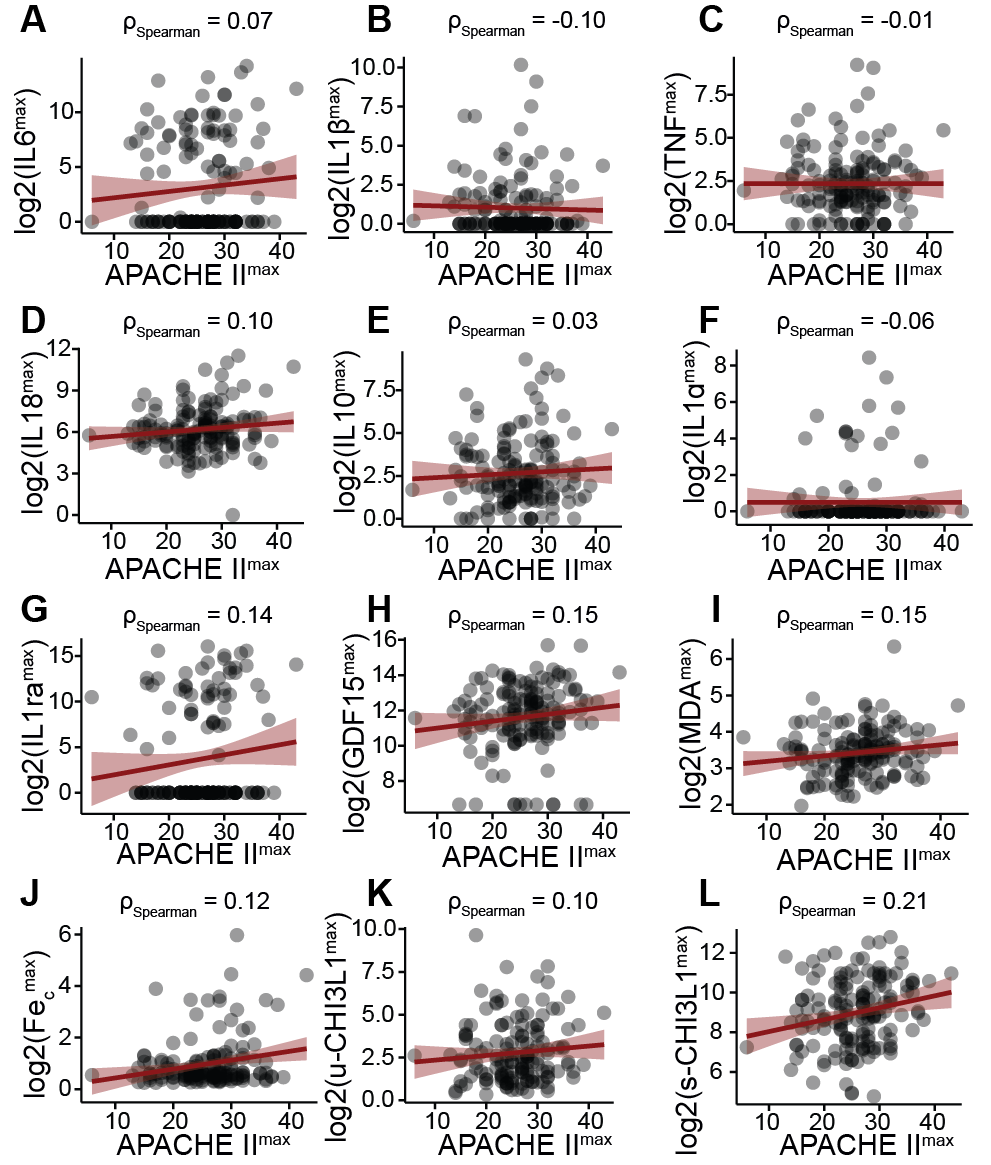


**Figure S3:** **Scatter plots and correlations between maximal biomarkers levels and APACHE II scores during the first seven days in the ICU.**

(A-L) Correlation between log2-transformed values of interleukin-6 (IL6), interleukin-1 beta (IL-1β), tumor necrosis factor (TNF), interleukin-18 (IL-18), interleukin-10 (IL-10), interleukin-1 alpha (IL-1α), interleukin-1 receptor antagonist (IL-1Ra), GDF15, free malondialdehyde (MDA), catalytic iron (Fe_c_), urinary chitinase-3-like protein 1 (u-CHI3L1), and serum chitinase-3-like protein 1 (s-CHI3L1) (highest value during the first seven days after ICU admission) and maximal APACHE II score attained during the first seven days after ICU admission.


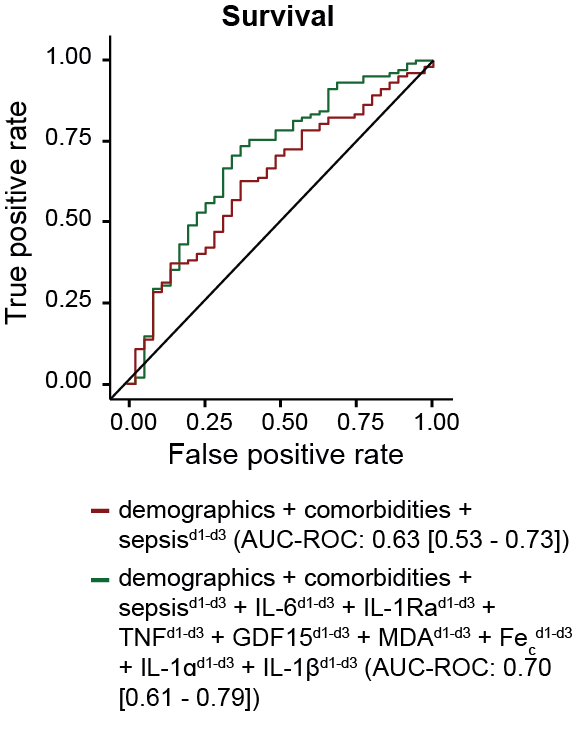


**Figure S4:** **Comparison of survival predictions when complementing demographics, comorbidities, and established sepsis diagnosis with biomarker levels**.

ROC curves for survival prediction comparing two models: one including demographics data, comorbidities, and established sepsis diagnosis (red), and an optimized model with additional variables, specifically maximal values during the first three days after ICU admission of a subset of biomarkers (green).


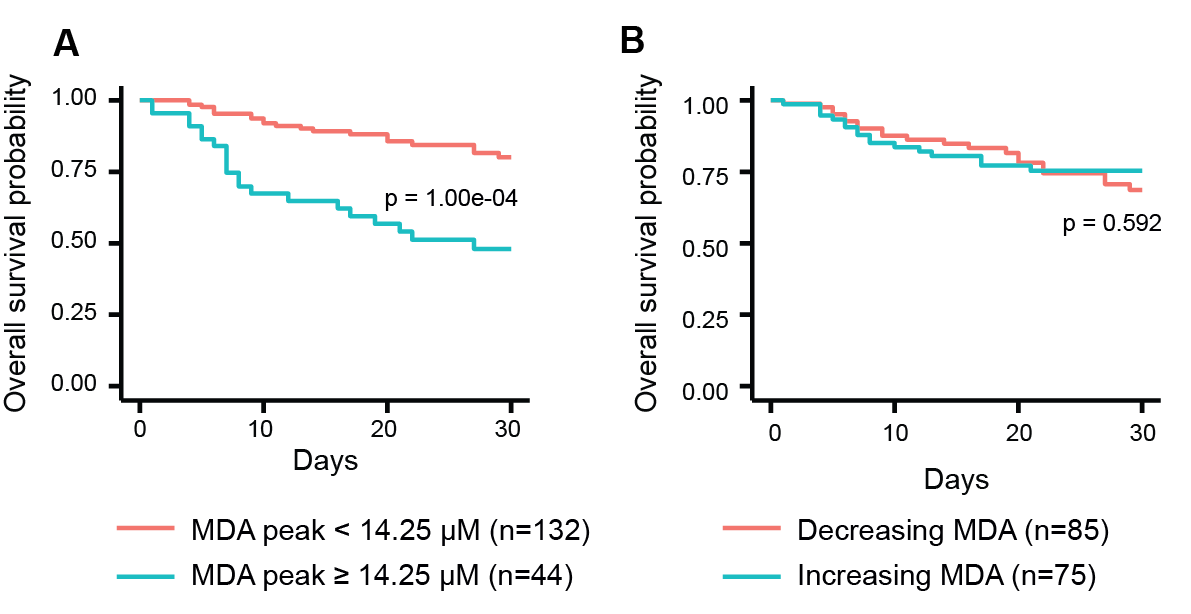


**Figure S5:** **Kaplan-Meier survival curves comparing the survival of MDA^+^ patients vs. MDA^-^ as defined by**:

(A) Patients with an MDA peak above 14.25 µM during the first 7 days in the ICU are MDA^+^.

(B) Patients belonging to the cluster with increasing MDA are MDA^+^ and those belonging to the cluster with decreasing MDA are MDA^-^. The p-value was calculated using the log-rank test.


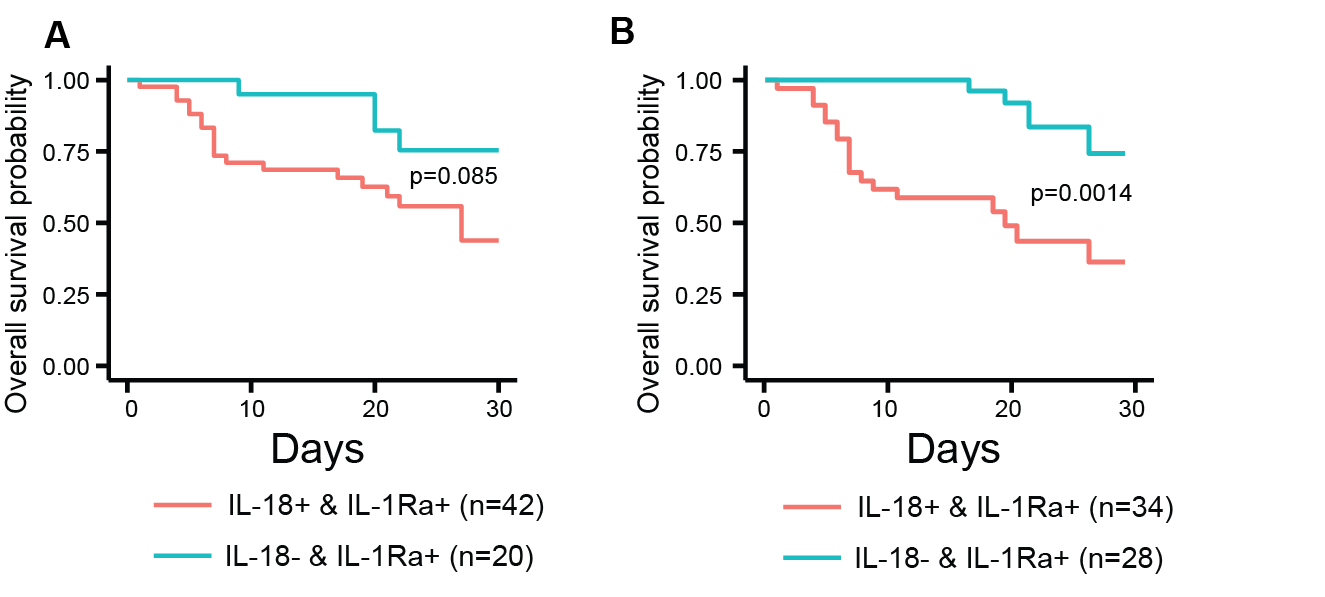


**Figure S6:** **Kaplan-Meier survival curves comparing the survival of IL-1Ra^+^/IL-18^+^ patients vs. IL-1Ra^+^/IL-18^-^ as defined by:**

(A) Patients with a detectable amount of IL-1Ra during the first 7 days in the ICU are IL-1Ra^+^ and they are IL-18^+^ if the IL-18 peaks above 100 pg/mL.

(B) Patients with a detectable amount of IL-1Ra during the first 7 days in the ICU are IL-1Ra^+^ and they are IL-18^+^ if belonging to cluster 2 of the IL-18 longitudinal clustering (i.e., increasing trend). The p-value was calculated using the log-rank test.


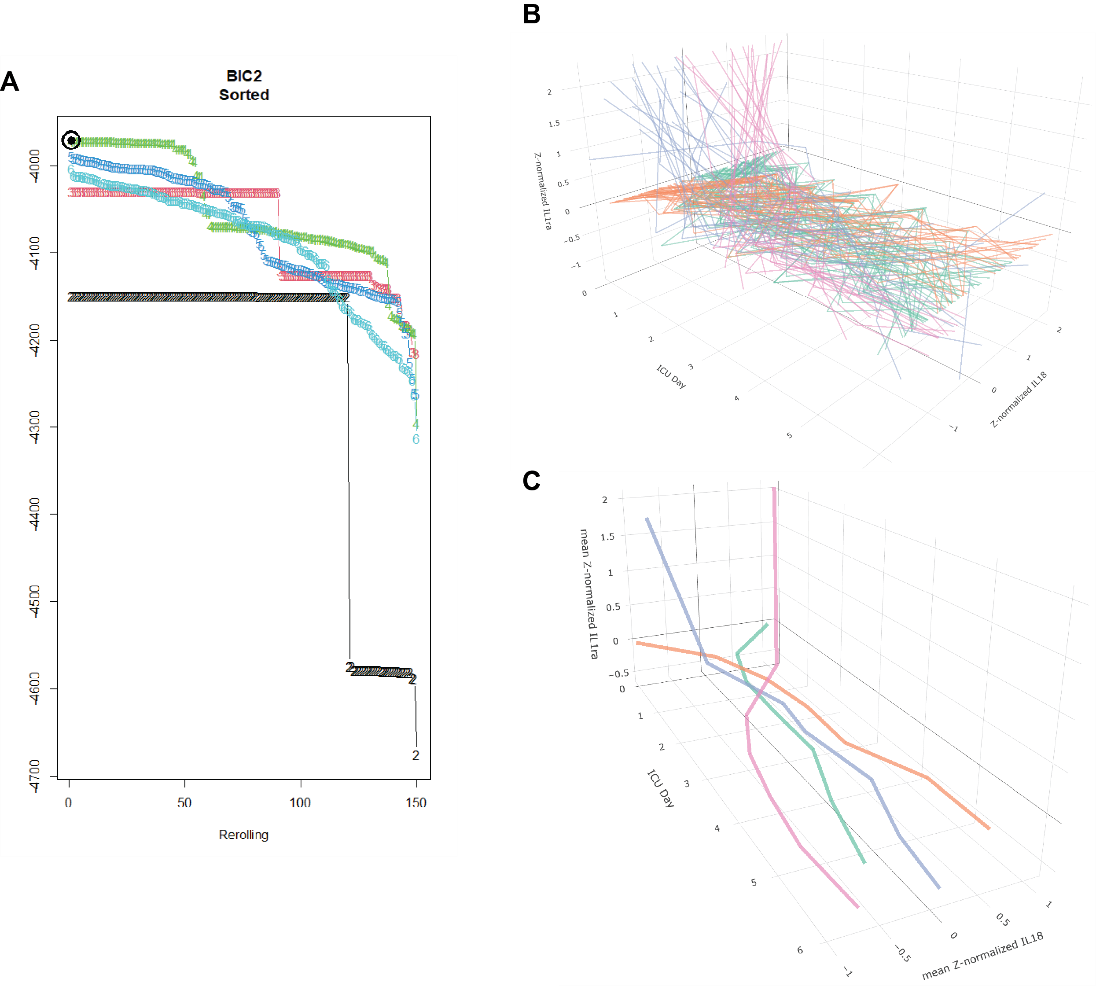


**Figure S7:** **Longitudinal clustering of IL-18 & IL-1Ra for trajectories beginning on IL-1Ra peaks.**

(A) Bayesian Information Criterion (BIC) for different rerolling of the clustering. The upper left point, corresponding to 4 clusters, was chosen.

(B) Trajectories of z-normalized values of IL-1Ra and IL-18 over the days. If an IL-1Ra peak happened later than day 1, the trajectory was shifted to the left to synchronize the peaks across patients.

(C) Mean trajectories of z-normalized values of IL-1Ra and IL-18 for the four clusters.


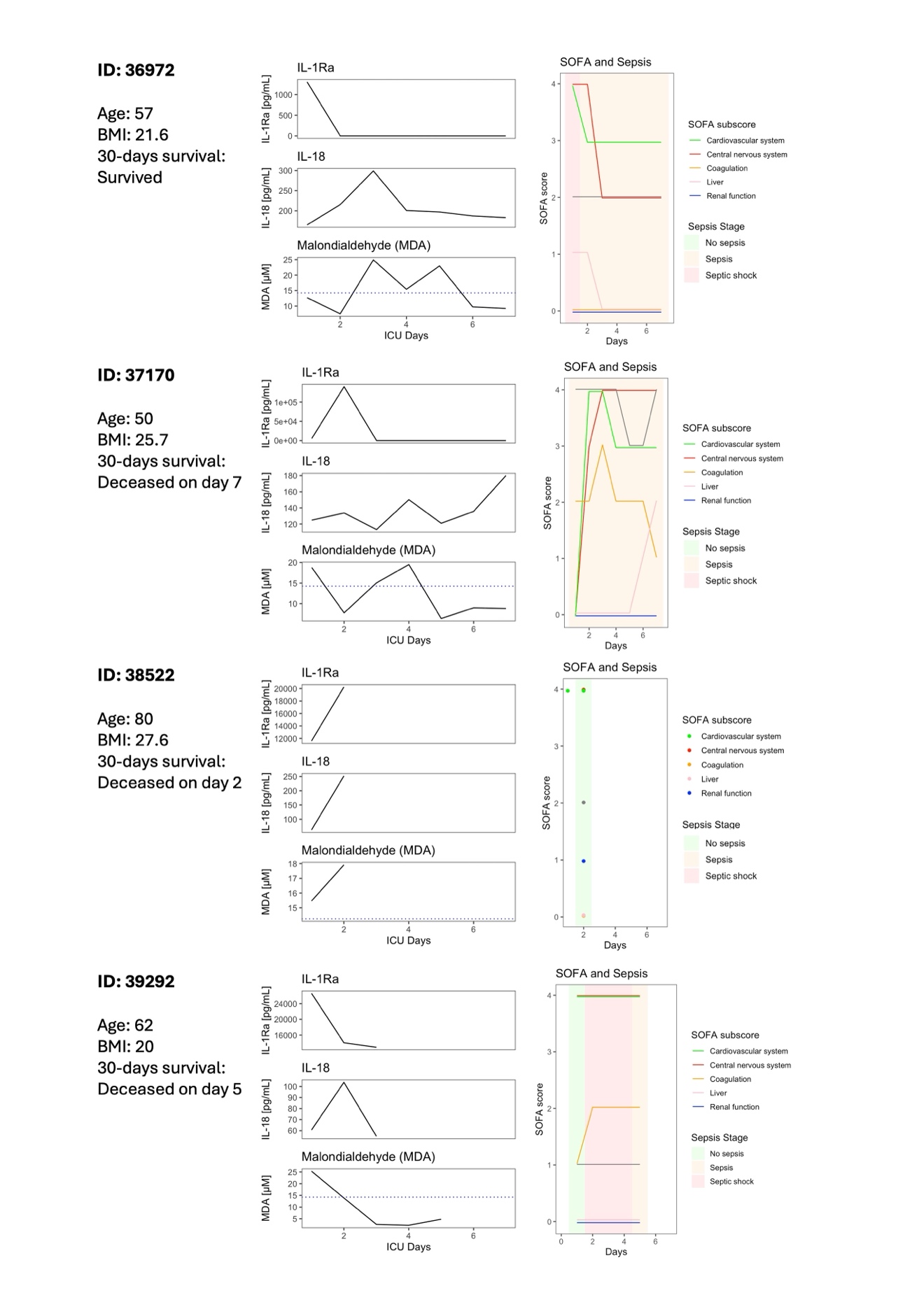
**Figure S8:** **Individual trajectories of IL-1Ra, IL-18, MDA, and SOFA scores in triple-positive patients.**

# Biomarker concentrations and SOFA scores are shown for each of the 14 patients classified as triple-positive (IL-1Ra⁺, IL-18⁺, and MDA⁺) during the first seven days of ICU admission. Each panel displays the temporal evolution of IL-1Ra, IL-18, MDA, and SOFA subscores for an individual patient.


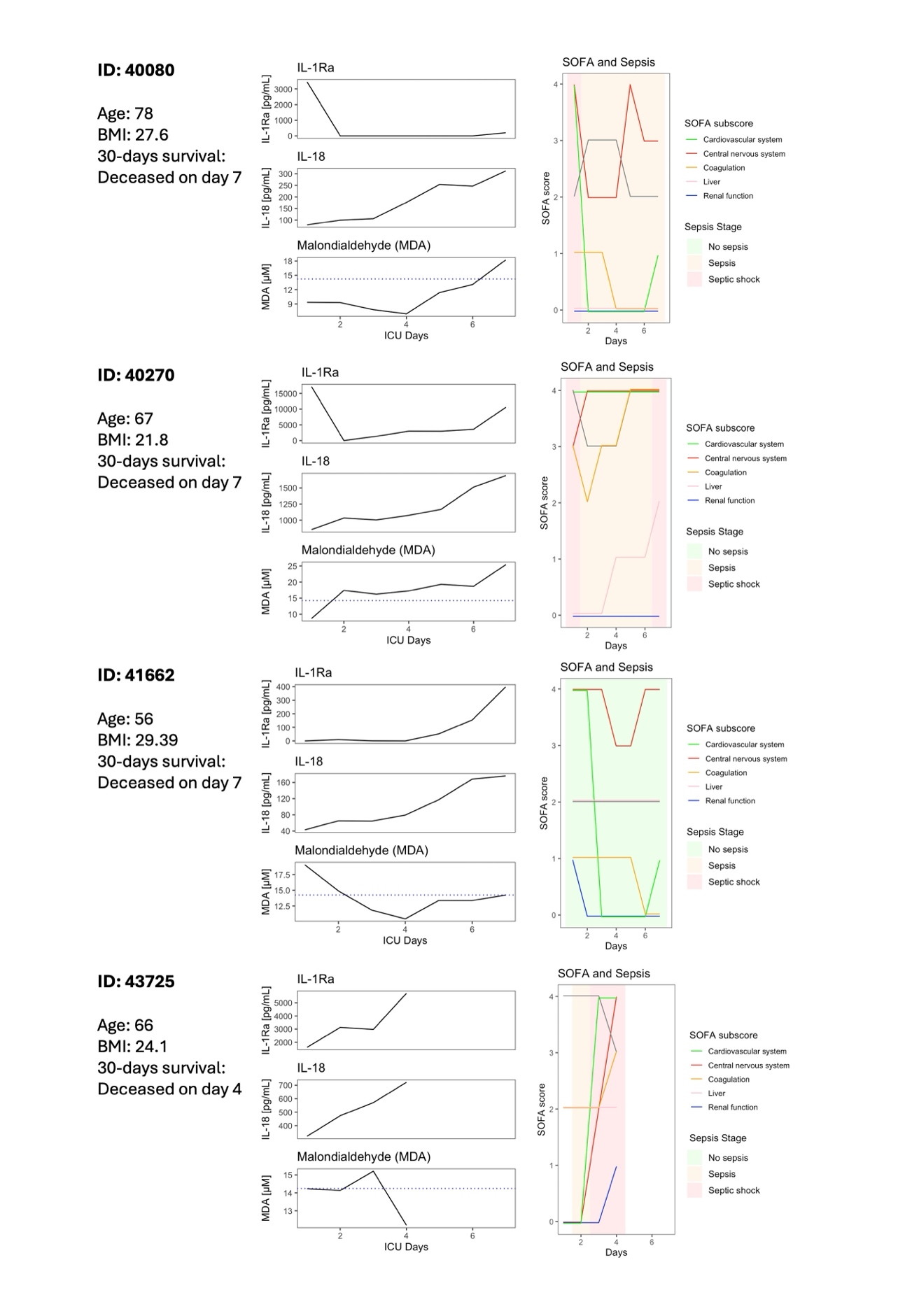
**Figure S8 (continued)**


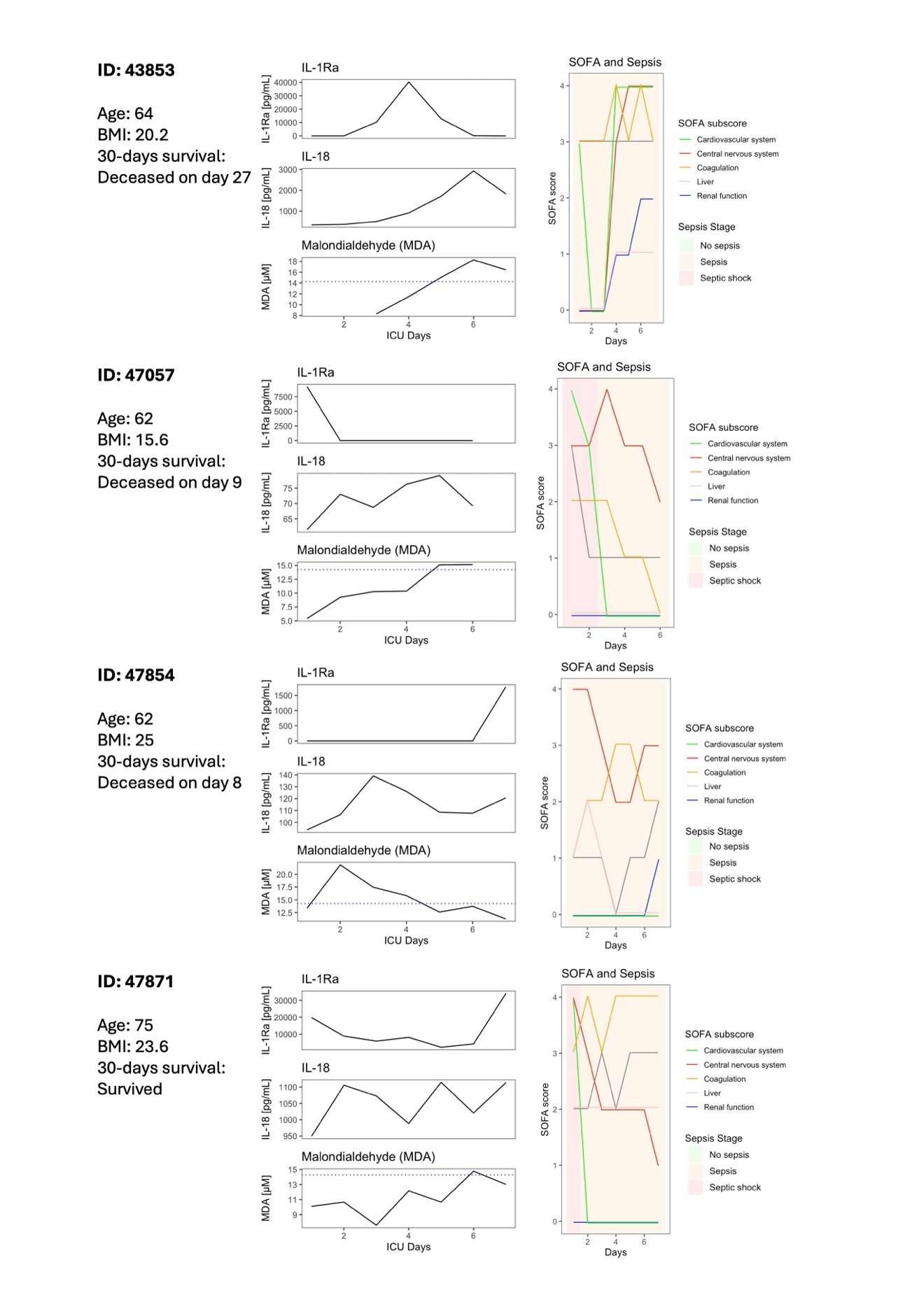
**Figure S8 (continued)**


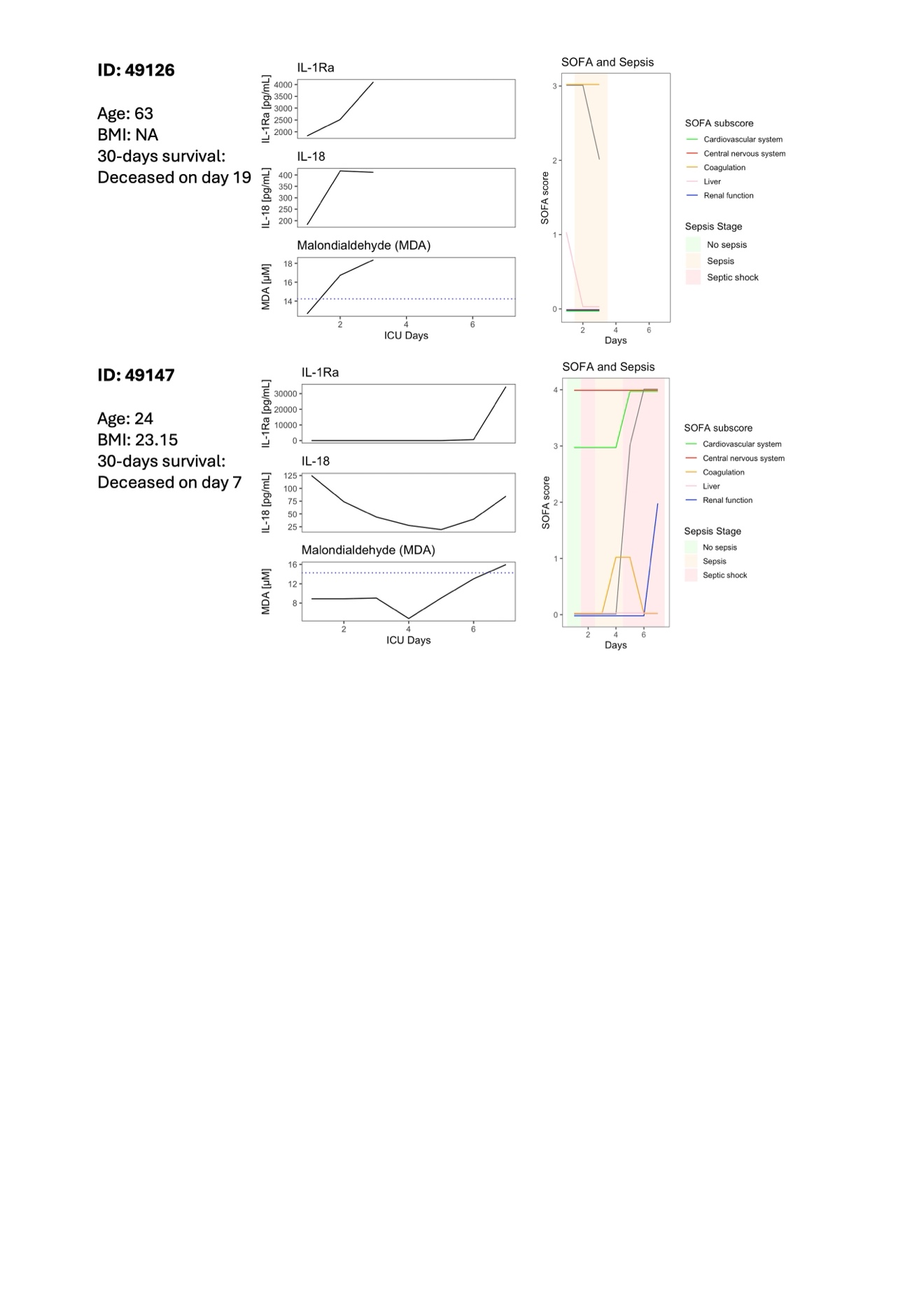


**Figure S8 (continued)**

# Supplementary Tables

**Table S1: Inclusion and exclusion criteria of the study**

| **Inclusion** | **Exclusion** |
| --- | --- |
| Age ≥ 18 yo | CKD KDOQI stage 5 (GFR <15 ml/min/1.73 m2 or RRT) |
| Presence of both arterial and urinary catheter |  |
| Expected ICU stay ≥48 h |  |
| Respiratory SOFA score ≥2 (PaO2/FiO2 < 300) or cardiovascular SOFA score ≥1 (MAP <70 mmHg or on vasopressor(s) for at least 1 h) |  |
| Written informed consent |  |

KDOQI: Kidney Disease Outcomes Quality Initiative, CKD: chronic kidney disease, FiO2: fraction of inspired oxygen, GFR: glomerular filtration rate, ICU: intensive care unit, MAP: mean arterial pressure, PaO2: partial pressure of oxygen in arterial blood, RRT: renal replacement therapy, SOFA: Sequential Organ Failure Assessment.

**Table S2: Overview of infection foci and isolated organisms.**

| **Focus of infection** | **No. of patients (% of patients with infection)** |
| --- | --- |
| Respiratory tract | 120 (88%) |
| Abdominal | 26 (19%) |
| Skin | 8 (6%) |
| Urinary tract | 7 (5%) |
| Catheter | 6 (4%) |
| Endocarditis | 3 (2%) |
| Cerebrospinal fluid | 3 (2%) |
| Other | 8 (6%) |
| Unknown | 43 (32%) |
| **Organisms isolated** | **No. of patients (% of patients with infection)** |
| *Escherichia coli* | 29 (21%) |
| *Pseudomonas aeruginosa* | 13 (10%) |
| *Klebsiella pneumoniae* | 12 (9%) |
| *Staphylococcus aureus* | 12 (9%) |
| *Haemophilus influenzae* | 9 (7%) |
| *Staphylococcus epidermidis* | 8 (6%) |
| Other | 84 (62%) |
| Unknown | 70 (51%) |

Multiple infection sites may be reported for a single patient.

Some patients may have multiple organisms isolated. ‘Unknown’ includes those with negative cultures as well as those whose culture results were unavailable.

**Table S3: Cox proportional hazards model investigating the association between biomarker levels and survival during the 30 days after ICU admission adjusted for demographics and comorbidities**.

| **Characteristic** | **HR** | **95% CI** | **p-value** |
| --- | --- | --- | --- |
| IL-6^max^ | 1.05 | 0.95, 1.16 | 0.57 |
| IL-1b^max^ | 1.05 | 0.71, 1.54 | 0.89 |
| TNF^max^ | 0.66 | 0.44, 1.01 | 0.16 |
| IL-18^max^ | 1.01 | 0.73, 1.39 | 0.97 |
| IL-10^max^ | 1.20 | 0.91, 1.58 | 0.39 |
| IL-1a^max^ | 0.82 | 0.61, 1.09 | 0.35 |
| IL-1Ra^max^ | 1.17 | 1.05, 1.30 | **0.02** |
| MDA^max^ | 1.83 | 1.16, 2.88 | **0.04** |
| Fe_c_^max^ | 1.20 | 0.84, 1.71 | 0.54 |
| GDF15^max^ | 0.85 | 0.68, 1.06 | 0.31 |
| s-CHI3L1^max^ | 0.96 | 0.70, 1.30 | 0.87 |
| u-CHI3L1^max^ | 0.89 | 0.67, 1.17 | 0.60 |
| Age | 1.04 | 1.01, 1.07 | **0.03** |
| BMI | 0.97 | 0.88, 1.06 | 0.68 |
| **Gender** |  |  |  |
| F |  |  |  |
| M | 0.72 | 0.35, 1.47 | 0.59 |
| **Heart failure** |  |  |  |
| No |  |  |  |
| Yes | 0.38 | 0.03, 4.31 | 0.62 |
| **Diabetes** |  |  |  |
| No |  |  |  |
| Yes | 0.31 | 0.06, 1.56 | 0.34 |

HR: Hazard ratio, CI: confidence interval, BMI: body mass index.

**Table S4: Cox proportional hazards model investigating the association between biomarker levels and survival during the 30 days after ICU admission adjusted for SOFA score, demographics and comorbidities.**

| **Characteristic** | **HR** | **95% CI** | **p-value** |
| --- | --- | --- | --- |
| IL-6^max^ | 1.06 | 0.94, 1.18 | 0.52 |
| IL-1b^max^ | 1.14 | 0.74, 1.75 | 0.72 |
| TNF^max^ | 0.62 | 0.38, 1.00 | 0.14 |
| IL-18^max^ | 1.03 | 0.73, 1.45 | 0.94 |
| IL-10^max^ | 1.32 | 0.98, 1.77 | 0.17 |
| IL-1a^max^ | 0.79 | 0.60, 1.04 | 0.22 |
| IL-1Ra^max^ | 1.13 | 1.00, 1.29 | 0.14 |
| MDA^max^ | 1.65 | 1.02, 2.66 | 0.12 |
| Fe_c_^max^ | 0.99 | 0.62, 1.59 | 0.94 |
| GDF15^max^ | 0.87 | 0.69, 1.08 | 0.39 |
| s-CHI3L1^max^ | 0.91 | 0.64, 1.28 | 0.72 |
| u-CHI3L1^max^ | 0.91 | 0.64, 1.28 | 0.72 |
| Age | 1.04 | 1.01, 1.07 | 0.06 |
| BMI | 0.97 | 0.88, 1.07 | 0.72 |
| **Gender** |  |  |  |
| F |  |  |  |
| M | 0.58 | 0.28, 1.24 | 0.39 |
| **Heart failure** |  |  |  |
| No |  |  |  |
| Yes | 0.29 | 0.02, 4.60 | 0.59 |
| **Diabetes** |  |  |  |
| No |  |  |  |
| Yes | 0.26 | 0.05, 1.37 | 0.25 |
| SOFA^max^ | 1.08 | 0.92, 1.27 | 0.52 |

HR: Hazard ratio, CI: confidence interval, BMI: body mass index, SOFA: sequential organ assessment

**Table S5: Cox proportional hazards model investigating the association between biomarker levels and survival during the 30 days after ICU admission adjusted for APACHE II score, demographics and comorbidities.**

| **Characteristic** | **HR** | **95% CI** | **p-value** |
| --- | --- | --- | --- |
| IL-6^max^ | 1.03 | 0.90, 1.18 | 0.75 |
| IL-1b^max^ | 1.18 | 0.74, 1.89 | 0.67 |
| TNF^max^ | 0.85 | 0.46, 1.57 | 0.73 |
| IL-18^max^ | 0.99 | 0.67, 1.46 | 0.97 |
| IL-10^max^ | 1.5 | 1.04, 2.16 | 0.09 |
| IL-1a^max^ | 0.59 | 0.35, 1.02 | 0.16 |
| IL-1Ra^max^ | 1.03 | 0.90, 1.18 | 0.78 |
| MDA^max^ | 1..61 | 0.96, 2.69 | 0.17 |
| Fe_c_^max^ | 0.85 | 0.52, 1.41 | 0.69 |
| GDF15^max^ | 0.81 | 0.63, 1.03 | 0.20 |
| s-CHI3L1^max^ | 0.94 | 0.67, 1.34 | 0.84 |
| u-CHI3L1^max^ | 1.14 | 0.83, 1.56 | 0.62 |
| Age | 1.01 | 0.97, 1.04 | 0.85 |
| BMI | 1.04 | 0.92, 1.17 | 0.68 |
| **Gender** |  |  |  |
| F |  |  |  |
| M | 0.55 | 0.23, 1.29 | 0.35 |
| **Heart failure** |  |  |  |
| No |  |  |  |
| Yes | 0.24 | 0.01, 3.91 | 0.54 |
| **Diabetes** |  |  |  |
| No |  |  |  |
| Yes | 0.44 | 0.07, 2.65 | 0.59 |
| APACHE II^max^ | 1.16 | 1.06, 1.28 | **0.01** |

HR: Hazard ratio, CI: confidence interval, BMI: body mass index, SOFA: sequential organ assessment

**Table S6: Cox proportional hazards model investigating the association between biomarker-based clusters and survival during the 30 days after ICU admission adjusted for SOFA score, demographics and comorbidities.**

| **Characteristic** | **HR** | **95% CI** | **p-value** |
| --- | --- | --- | --- |
| **Cluster** |  |  |  |
| 2-on-1 | 2.04 | 0.71, 5.89 | 0.39 |
| 3-on-1 | 0.73 | 0.24, 2.25 | 0.72 |
| 4-on-1 | 1.76 | 0.67, 4.62 | 0.52 |
| 5-on-1 | 1.77 | 0.36, 8.75 | 0.67 |
| Age | 1.03 | 1.01, 1.06 | **0.04** |
| BMI | 0.95 | 0.87, 1.04 | 0.52 |
| **Gender** |  |  |  |
| F |  |  |  |
| M | 0.51 | 0.26, 1.02 | 0.16 |
| **Diabetes** |  |  |  |
| No |  |  |  |
| Yes | 0.55 | 0.12, 2.41 | 0.59 |
| **Heart failure** |  |  |  |
| No |  |  |  |
| Yes | 1.04 | 0.14, 7.95 | 0.94 |
| SOFA^d1^ | 1.07 | 0.95, 1.20 | 0.52 |

HR: Hazard ratio, CI: confidence interval, BMI: body mass index, SOFA: sequential organ assessment

**Table S7: Cox proportional hazards model investigating the association between biomarker-based clusters and survival during the 30 days after ICU adjusted for demographics and comorbidities.**

| **Characteristic** | **HR** | **95% CI** | **p-value** |
| --- | --- | --- | --- |
| **Cluster** |  |  |  |
| 2-on-1 | 1.68 | 0.61, 4.63 | 0.52 |
| 3-on-1 | 0.81 | 0.28, 2.33 | 0.82 |
| 4-on-1 | 2.08 | 0.84, 5.17 | 0.25 |
| 5-on-1 | 1.88 | 0.39, 9.04 | 0.59 |
| Age | 1.03 | 1.01, 1.06 | **0.05** |
| BMI | 0.96 | 0.88, 1.05 | 0.59 |
| **Gender** |  |  |  |
| F |  |  |  |
| M | 0.61 | 0.31, 1.21 | 0.39 |
| **Diabetes** |  |  |  |
| No |  |  |  |
| Yes | 0.58 | 0.13, 2.52 | 0.67 |
| **Heart failure** |  |  |  |
| No |  |  |  |
| Yes | 1.04 | 0.14, 7.77 | 0.94 |

HR: Hazard ratio, CI: confidence interval, BMI: body mass index
